# Perinatal Propionate Supplementation Protects Adult Male Offspring from Maternal Chronic Kidney Disease-Induced Hypertension

**DOI:** 10.3390/nu14163435

**Published:** 2022-08-21

**Authors:** You-Lin Tain, Chih-Yao Hou, Guo-Ping Chang-Chien, Su-Fan Lin, Chien-Ning Hsu

**Affiliations:** 1 Department of Pediatrics, Kaohsiung Chang Gung Memorial Hospital, Kaohsiung 833, Taiwan; 2 College of Medicine, Chang Gung University, Taoyuan 330, Taiwan; 3 Department of Seafood Science, National Kaohsiung University of Science and Technology, Kaohsiung 811, Taiwan; 4 Institute of Environmental Toxin and Emerging-Contaminant, Cheng Shiu University, Kaohsiung 833, Taiwan; 5 Super Micro Mass Research and Technology Center, Cheng Shiu University, Kaohsiung 833, Taiwan; 6 Center for Environmental Toxin and Emerging-Contaminant Research, Cheng Shiu University, Kaohsiung 833, Taiwan; 7 Department of Pharmacy, Kaohsiung Chang Gung Memorial Hospital, Kaohsiung 833, Taiwan; 8 School of Pharmacy, Kaohsiung Medical University, Kaohsiung 807, Taiwan

**Keywords:** gut microbiota, short-chain fatty acid, developmental origins of health and disease (DOHaD), propionate, hypertension, chronic kidney disease, inflammation

## Abstract

Emerging evidence supports that early-life disturbance of gut microbiota has an impact on adult disease in later life. Offspring hypertension can be programmed by maternal chronic kidney disease (CKD). Conversely, perinatal use of gut microbiota-targeted therapy has been implemented to reverse programming processes and prevent hypertension. Short-chain fatty acids (SCFAs), the major gut microbiota-derived metabolites, can be applied as postbiotics. Propionate, one of predominant SCFAs, has been shown to have antihypertensive property. We examined whether perinatal propionate supplementation can prevent offspring hypertension induced by maternal CKD. CKD was induced by chow supplemented with 0.5% adenine for 3 weeks before pregnancy. Propionate (P) was supplemented at 200 mmol/L in drinking water during pregnancy and lactation. Male offspring were divided into four groups (*n* = 7–8/group): control, CKD, control+propionate (CP), and CKD+propionate (CKDP). Maternal CKD-induced offspring hypertension was reversed by perinatal propionate supplementation. The protective effects of perinatal propionate treatment were related to increased propionate-generating bacteria *Clostridium* spp. and plasma propionate level, increased expression of renal G protein-coupled receptor 41 (GPR41, a SCFA receptor), augmentation of α-diversity, and shifts in gut microbiota composition. In summary, our results highlight that maternal CKD-induced offspring hypertension can be prevented by the use of gut microbial metabolite SCFAs in early life, which could shed light on the prevention of the current hypertension pandemic.

## 1. Introduction

There are trillions of bacteria in the gut microbiota that influence human health [1]. Perturbation in the composition and function of the gut microbiota, termed gut dysbiosis, has been associated with many diseases. Maternal and fetal exposures to environmental insults have been associated with disruption of the offspring gut microbiota, which precedes later onset of disease in adult life [2]. Various pre-, peri-, and early postnatal factors govern the establishment of the gut microbiota, such as maternal illness, delivery methods, gestational age, formula feeding, antibiotic exposure, and ecological factors [2,3].

Chronic kidney disease (CKD) is closely linked to adverse maternal and fetal outcomes and is reported to affect at least 3%–4% women of childbearing age [4,5]. In CKD, gut dysbiosis can contribute to CKD progression and CKD-related comorbidities through diverse mechanisms involving microbiota-derived metabolites [6]. Using a maternal adenine-induced CKD model, we previously found that adult male progeny developed hypertension accompanied by abnormalities of the gut microbiota and their derived metabolites [7].

Microbial metabolite short-chain fatty acids (SCFAs) are generally known to induce vasodilatation, in favor of antihypertension [8]. Circulating SCFAs exert their regulatory function on blood pressure (BP) by activating their receptors in the kidneys and interfering with key factors of the kidney–gut axis [9,10]. Propionate, one of the predominant SCFAs, has been shown to have benefits on human health [11]. Additionally, aberrant immune responses and renal inflammation are involved in CKD and hypertension, which can be modulated by SCFAs [12,13].

Increasing evidence supports that early-life gut microbiota-targeted therapies have the potential to prevent the development of CKD and its comorbidities later in life [13]. Similar to probiotics and prebiotics, postbiotics (metabolic bioproducts generated by probiotics) also contribute to the improvement of host health [14]. Prior work indicates that SCFAs acting as postbiotics are able to avert hypertension of developmental origins [15,16]. However, the impact of perinatal propionate supplementation on maternal CKD-induced offspring hypertension remains unknown. Our previous report showed that mother rats fed with 0.5% adenine 3 weeks before mating developed CKD, which was characterized by renal dysfunction (~30% of normal renal function), glomerular and tubulointerstitial injuries, and hypertension [7]. Besides, adenine-induced CKD diminished fecal propionate concentrations and the proportion of propionate-producing microbe in mother rats. Hence, the aim of this study is to evaluate whether perinatal propionate supplementation can avert offspring hypertension induced by maternal CKD through shifts in the microbial compositions of gut microbiota, alterations of the SCFA signaling pathway, and reduction in renal inflammation.

## 2. Materials and Methods

### 2.1. Animals and Study Design

Female Sprague–Dawley (SD) rats aged 8 weeks were purchased from BioLASCO Taiwan Co., Ltd. (Taipei, Taiwan). Rats were housed in our animal facility granted a full accreditation by the Association for Assessment and Accreditation of Laboratory Animal Care International (AAALAC). The procedures used in this study were in agreement with the rules of the IACUC of Chang Gung Memorial Hospital (Permit # 2021031705) and the Care and Use of Laboratory Animals of the National Institutes of Health.

**Figure 1:**
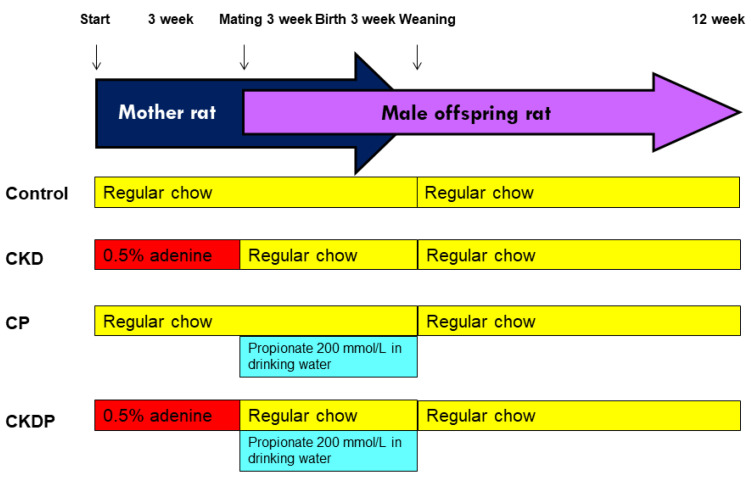
Experimental protocol used in the current study. CKD = adenine-treated rats; CP = control rats received propionate; CKDP = adenine-treated rats received propionate.

Figure 1 illustrates the experimental protocol. To conduct a CKD model, 8-week-old female rats received chow supplemented with 0.5% adenine for 3 weeks, as stated before [7]. At 11 weeks of age, female rats were caged with male rats until mating. Mating was confirmed by the presence of a copulatory plug. A total of 12 dams were randomly divided into four groups (n = 3 per group): control, CKD (adenine-treated rats), CP (control rats received propionate supplemented at 200 mmol/L in drinking water during gestation and lactation), and CKDP (adenine-treated rats received propionate supplemented at 200 mmol/L in drinking water during gestation and lactation). The dose of propionate used here are according to prior work conducted in rodents [17]. After birth, litter size was standardized to eight pups. As males were counted as having hypertension earlier than females [18], we only recruited male offspring from each litter (n = 2–3 per dam) for use in subsequent experiments. A total of four experimental groups were studied (n = 7–8 per group): C, CKD, CP, and CKDP. At 3 weeks old, pups were weaned and put onto regular chow.

According to our protocol [7], rats were acclimated to the CODA rat tail-cuff system (Kent Scientific Corporation, Torrington, CT, USA) to check BP every month. Offspring rats were killed at 12 weeks of age. Prior to sacrifice, stool samples were collected in the morning and stored at −80 °C in a freezer until analysis. Blood samples were collected in a heparin tube. The kidneys were collected, divided into cortex and medulla, and snap-frozen at −80 °C until analysis.

### 2.2. Gas Chromatography–Mass Spectrometry (GC–MS)

We implemented a gas chromatograph–mass spectrometer (Agilent Technologies, Wilmington, DE, USA) to measure plasma levels of SCFAs, including acetic acid, propionic acid, isobutyric acid, butyric acid, isovaleric acid, and valeric acid. The chromatographic separation was performed on a DB-FFAP column (30 cm × 0.25 mm, 0.25 µm; Agilent Technologies) with an injection port temperature of 240 °C [19]. Injections were held with a volume of 1 µL and a split ratio of 5:1. We used 2-ethylbutyric acid as an internal standard.

### 2.3. Measurement of Cytokines in the Offspring Kidneys

We used a LEGENDplex^TM^ multiplex cytokine panel kit (BioLegend, San Diego, CA, USA) and a BD FACSCanto II flow cytometer (BD Biosciences, San Jose, CA, USA) to determine cytokine levels in the renal cortical protein extracts. These cytokines include interferon-γ (IFN-γ), interleukin (IL)-1A, IL-1B, IL-2, IL-6, and IL-17A. The fluorescent intensity of the target cytokine level was determined in each bead set by its fluorescent color code. Data generated by a flow cytometer was quantified by LEGENDplex analysis software (BioLegend). All samples were measured in duplicate.

### 2.4. Quantitative PCR

RNA was extracted from rat kidney cortex tissue as stated before [7]. Several SCFA receptors were determined, including G protein-coupled receptor 41 (GPR41), GPR43, GPR109A, and olfactory receptor 78 (Oflr78). Quantitative PCR was performed using the QuantiTect SYBR Green PCR Reagents kit (Qiagen, Santa Clarita, CA, USA) on the iCycler iQ Real-Time PCR Detection System (Bio-Rad, Hercules, CA, USA). The R18S reference gene was used as the internal control. The primers were designed using GeneTool Software (BioTools, Edmonton, AB, Canada). Table 1 provides the PCR primer sequences. Each sample was run in duplicate. To determine relative gene expression, the comparative threshold cycle (Ct) method was used. The fold increase in the experimental sample, relative to the control, was calculated using the formula 2^−ΔΔCt^.

### 2.5. 16S rRNA Gene Sequencing

As stated before [7,15], total genomic DNA from fecal specimens was extracted and analyzed by 16S rRNA by metagenomics analysis at Biotools Co., Ltd. (Taipei, Taiwan). The full-length 16S genes (V1–V9 regions) were amplified with barcoded 16S gene-specific primers for multiplexed SMRTbell library preparation and sequencing procedure (PacBio, Menlo Park, CA, USA). The sequences were processed using QIIME (version 2, Knight Lab, Boulder, CO, USA). A phylogenetic tree was conducted with a set of sequences representative of the amplicon sequence variants (ASVs) using the QIIME2 phylogeny FastTree. We investigated the patterns of bacterial α- and β-diversity. Community richness was assessed by Faith’s phylogenetic diversity (PD) index, and the evenness accounting for the α-diversity was evaluated by the Shannon index. We evaluated the β-diversity of the gut microbiota across groups using principal coordinate analysis (PCoA) with unweighted UniFrac distance and the analysis of similarities (ANOSIM). The key bacterial taxa responsible for discrimination between different groups were determined using the LEfSe algorithm. The threshold on logarithmic score (LDA) for discriminative features was set to 4.

### 2.6. Statistical Analysis

Data are expressed as mean ± the standard error of the mean. Comparisons within groups were analysis using one-way ANOVA or two-way ANOVA where appropriate, followed by a Tukey’s post hoc test. A *p*-value less than 0.05 was considered statistically significant for all tests. Statistical analyses were performed using SPSS software (SPSS Inc., Chicago, IL, USA). Bioinformatics analyses of gut microbiota were carried out using R software (R core team, Vienna, Austria).

## 3. Results

### 3.1. Body Weight and Blood Pressure

The pup mortality rate was zero (Table 2). The body weight (BW) was lower in the CKDP group compared with the CP group. The kidney weight (KW) of the CKD group was higher than those of the C and CKDP groups, whereas the KW-to-BW ratio was comparable among the four groups.

**Figure 2:**
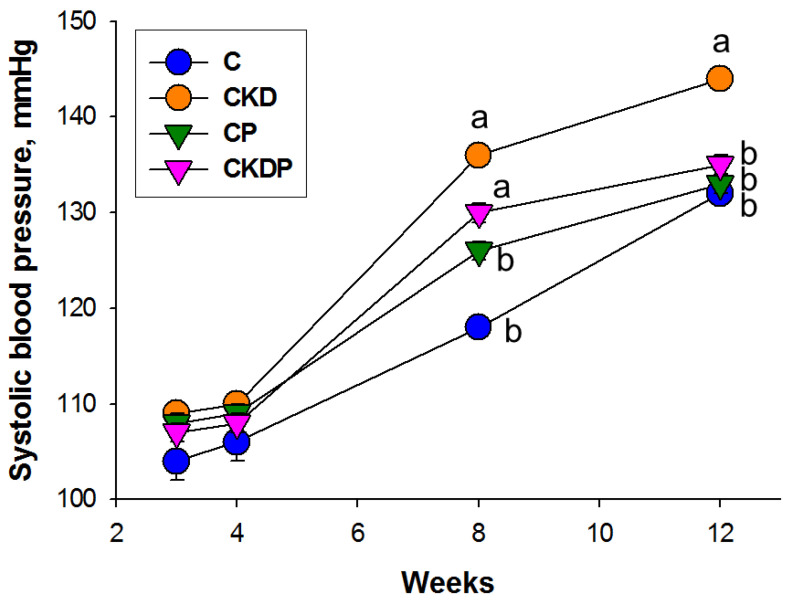
Effect of maternal CKD and propionate (P) supplementation on systolic blood pressure in offspring from 3 to 12 weeks of age. The letters a and b show significant differences between groups (*p* < 0.05, two-way ANOVA); N = 7–8/group. C = control; CKD = adenine-treated rats; CP = control rats received propionate; CKDP = adenine-treated rats received propionate.

Longitudinal measurement of BP between week 3 and week 12 revealed that maternal CKD caused increases in systolic BP (SBP) from 8 to 12 weeks of age, which was reversed by perinatal propionate supplementation (Figure 2). Likewise, mean arterial pressure was greater in the CKD group than the other groups in 12-week-old male offspring (Table 2). Taken together, these observations demonstrated that CKD resulted in hypertension in adult offspring, which perinatal propionate supplementation prevented.

### 3.2. Plasma SCFA Levels and SCFA Receptors

We first measured plasma SCFA levels and their receptors in offspring kidneys in response to maternal CKD and propionate supplementation. Table 3 illustrates that perinatal propionate supplementation caused an increase in plasma propionate level in the CP and CKDP group. Additionally, both maternal CKD and propionate supplementation had a negligible effect on acetic acid, butyric acid, isobutyric acid, isovaleric acid, and valeric acid in the plasma.

**Figure 3:**
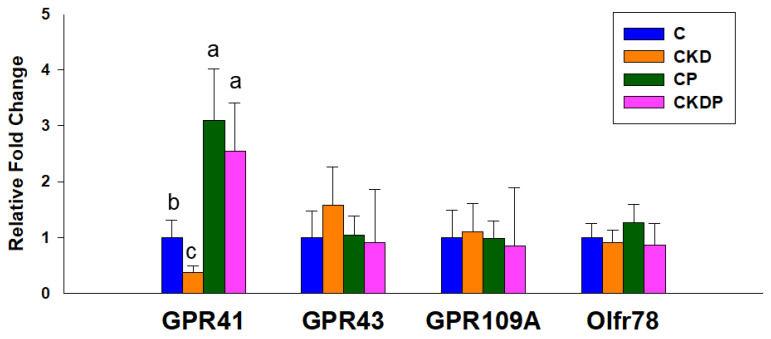
Effect of maternal chronic kidney disease (CKD) and propionate supplementation on the short-chain fatty acid receptor G protein-coupled receptor 41 (GPR41), GPR43, GPR91, and olfactory receptor 78 (Oflr78) in offspring kidneys at 12 weeks of age. The letters a, b, and c show significant differences between groups (*p* < 0.05, one-way ANOVA); N = 7–8/group. C = control; CKD = adenine-treated rats; CP = control rats received propionate; CKDP = adenine-treated rats received propionate.

We then determined the renal expression of SCFA receptors. Figure 3 illustrates that maternal CKD remarkably reduced renal GPR41 mRNA expression, which propionate supplementation prevented. In addition, maternal CKD or propionate supplementation had a negligible effect on renal GPR43, GPR91, and Oflr78 expression in adult offspring.

### 3.3. Cytokine Concentrations in the Kidneys

**Figure 4:**
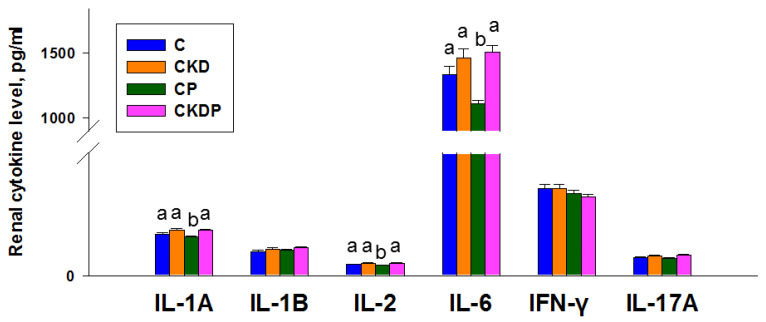
Effect of maternal CKD and propionate (P) supplementation on renal cytokine levels in offspring at 12 weeks of age. The letters a and b above the bars show significant differences between groups (*p* < 0.05, one-way ANOVA); N = 7–8/group. C = control; CKD = adenine-treated rats; CP = control rats received propionate; CKDP = adenine-treated rats received propionate.

We determined several cytokine levels in the offspring kidneys, including IL-1A, IL-1B, IL-2, IL-6, IFN-γ, and IL-17A. As shown in Figure 4, the renal levels of IL-1A, IL-2, and IL-6 were lowest in the CP groups compared with other groups. In addition, renal IL-1B, IFN-γ, and IL-17A levels were not different among the four groups.

### 3.4. Gut Microbiota Composition

**Figure 5:**
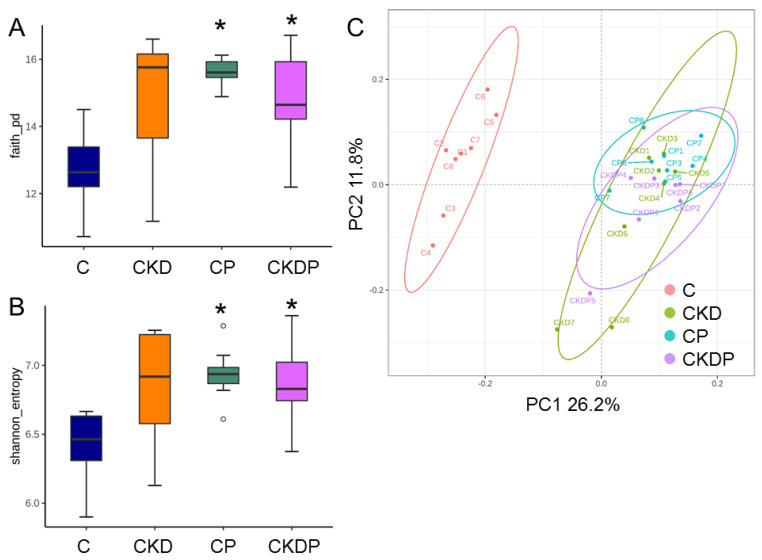
Comparison of bacterial α-diversity across four groups of offspring at the age of 12 weeks in (**A**) Faith’s phylogenetic diversity (PD) index and (**B**) Shannon index; (**C**) principal coordinate analysis (PCoA) based on unweighted UniFrac distance of the OTUs in four groups. Each point represents the microbiota of a single sample, and colors reflect metadata for that sample. * *p* < 0.05 vs. C.

To identify the effect of maternal CKD and propionate supplementation on the establishment of offspring gut microbiota, we analyzed α- and β-diversity metrics. Community richness (represented by Faith’s PD index) and evenness (represented by the Shannon index) were enhanced by propionate supplementation in the CP and CKDP groups compared with the C group (Figure 5A,B). As shown in Figure 5C, PCoA based on an unweighted UniFrac metric illustrated that the samples clustered according to study groups, and indicated that significant differences existed between the groups by ANOSIM (All *p* < 0.05). These data indicate that perinatal propionate supplementation caused shifts in offspring gut microbiota. Additionally, we found that *Firmicutes* and *Bacteroidetes* were the most predominant phyla, followed by *Actinobacteria* and *Proteobacteria*; our data were consistent with previous animal studies that gut microbiota is dominated by these bacteria [6,7].

**Figure 6:**
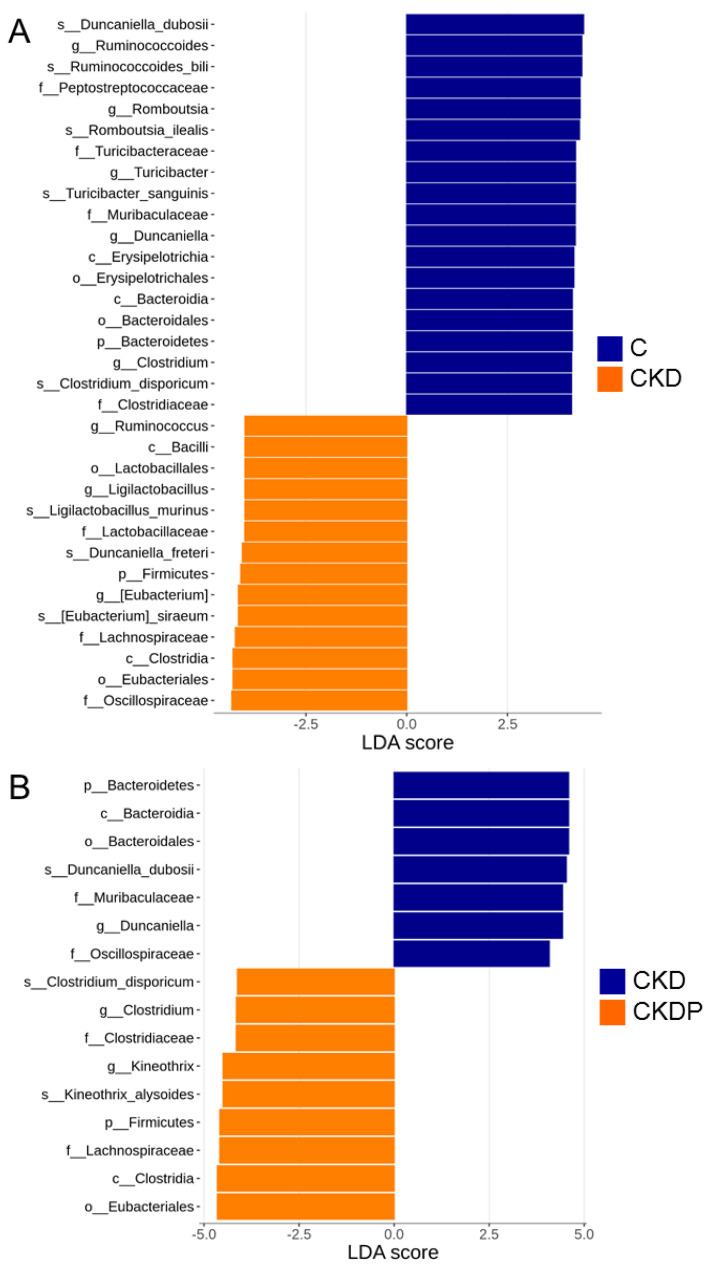
Linear discriminant analysis effect size (LEfSe) showing the most differentially abundant bacterial taxa between (**A**) the C and CKD group and (**B**) the CKD and CKDP group of offspring at the age of 12 weeks. The linear discriminant analysis (LDA) score threshold was set to greater than 4.

We also compared the gut microbiota in four study groups using LEfSe to identify the specific bacterial taxa associated with CKD and propionate supplementation. The greatest differences in multiple levels of taxa between the C and CKD group are shown in Figure 6A. The results revealed that the significantly increased genera *Ruminococcus*, *ligilactobacillus*, and *Eubacterium* can be one of the biomarkers of the CKD group. In contrast, maternal CKD resulted in a lower proportion of the genera *Romboutsia*, *Turicibacter*, *Duncaniella*, and *Clostridium*.

Figure 6B shows that propionate supplementation causes a higher genus level of *Clostridium* and *Kineothrix* in the CKDP group. We also found that the genus *Duncaniella* revealed a significant difference between the CKD and CKDP group; the CKDP group has a significantly lower level of *Duncaniella*.

**Figure 7:**
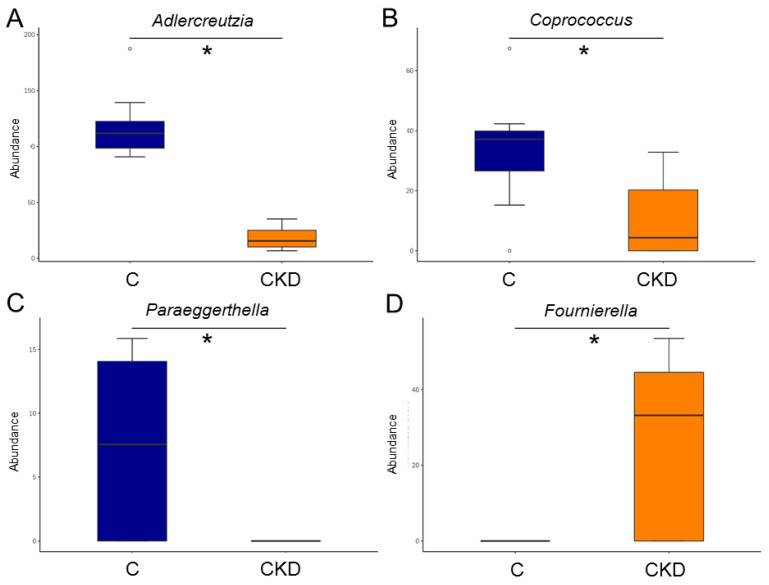
Effect of maternal CKD on the gut microbiota in 12-week-old male offspring. Genus-level relative abundance of (**A**) *Adlercreutzia*, (**B**) *Coprococcus*, (**C**) *Paraeggerthella,* and (**D**) *Fournierella*. * *p* < 0.05.

At the genus level, the abundances of *Adlercreutzia*, *Coprococcus*, and *Paraeggerthella* were reduced by maternal CKD (Figure 7A–C). The CKD group had a higher genus level of *Fournierella* in the CKD group vs. the C group (Figure 7D).

**Figure 8:**
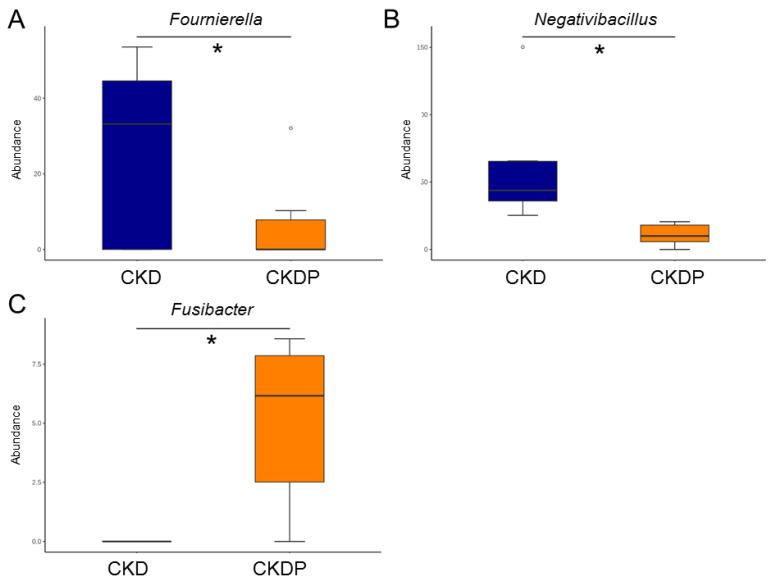
Effect of perinatal propionate supplementation on the gut microbiota in 12-week-old male offspring. Genus-level relative abundance of (**A**) *Fournierella*, (**B**) *Negativibacillus*, and (**C**) *Fusibacter*. * *p* < 0.05.

Propionate supplementation resulted in a reduction in the genus level of *Fournierella* and *Negativibacillus* in the CKDP group compared with the CKD group (Figure 8A,B). Conversely, propionate supplementation increased the abundance of *Fusibacter* in the CKDP group vs. the CKD group (Figure 8C).

## 4. Discussion

Our data provide novel evidence that propionate supplementation during pregnancy and lactation prevented offspring hypertension programmed by maternal CKD. The beneficial effects of perinatal propionate treatment were concurrent with increases in plasma propionate level, upregulation of renal GPR41 expression, shifts of gut microbiota, and augmentation of α-diversity. These results suggest that the use of postbiotic propionate in early life protects adult offspring against hypertension attributed to altered gut microbiota and their metabolites.

Growing evidence indicates adverse maternal conditions resulting in long-term negative effects on the offspring outcomes, including hypertension [20,21]. In support of our prior study in maternal adenine-induced CKD model [7], adult offspring born to mothers with CKD developed hypertension. We found that maternal CKD-induced offspring hypertension coincided with decreased GPR41 expression in offspring kidneys. Propionate is a ligand for GPR41, which has an antihypertensive effect [22]. Accordingly, the programming effects of maternal CKD-induced hypertension might be associated with its regulation on SCFA receptors to tip the balance between vasodilation and vasoconstriction in favor of the latter.

Another mechanism behind maternal CKD-induced hypertension might be due to the shift in gut microbiota compositions. Maternal CKD resulted in a particular reduction in the genera *Adlercreutzia*, *Clostridium*, and *Romboutsia*, whereas it increased genus *Fournierella* in the CKD group, which corresponded to elevated BPs. The present results support the notion of changes in the relative abundance of certain bacteria associated with hypertension, as previously reported [23,24]. Additionally, maternal CKD caused a trend for an increased proportion of the genera *Ruminococcus* and *Eubacterium*. Both *Ruminococcus* and *Eubacterium* have been reported to drive gut barrier dysfunction and intestinal inflammation, which correlates with increased BP [25].

To our knowledge, no prior studies have examined whether perinatal propionate treatment reverses the elevation of BP induced by maternal CKD in adult male progeny. The present results tie well with prior studies revealing that SCFAs could be used as a gut microbiota-targeted therapy to prevent offspring hypertension [15,16,26]. Belonging to SCFAs, propionate acting as a postbiotic has been shown to have benefits on human health [11]. Perinatal propionate supplementation prevented offspring hypertension, accompanied by shaping gut microbiota with increases in species richness and evenness, increasing the plasma propionate level and upregulating the renal GPR41 expression. The present results reveal the feasibility of manipulation of the gut microbiota by altering their metabolites by the early-life use of propionate to prevent offspring hypertension in later life.

Several kinds of intestinal microbes have been associated in the synthesis of propionate, such as *Ruminococcus*, *Eubacterium*, *Coprococcus*, and *Clostridium* [27]. Our data demonstrated that maternal CKD both increased (e.g., *Ruminococcus* and *Eubacterium*) and decreased (e.g., *Coprococcus* and *Clostridium*) certain propionate-producing bacteria, but in the end, had a negligible effect on plasma propionate level. On the other hand, propionate supplementation increased propionate-generating bacteria *Clostridium* spp. and the plasma propionate level. Furthermore, propionate treatment enhanced renal GPR41 expression. Therefore, the overall effects of propionate on SCFA production and its actions on SCFA receptors might be a major mechanism contributing to its benefit against offspring hypertension in this model.

Moreover, we found that propionate supplementation increased the abundance of *Fusibacter* but reduced the genera *Fournierella* and *Negativibacillus*. So far, very little is known about their relationships with hypertension of developmental origins. *Fusibacter* is a sulfur-reducing bacterium [28]. Considering that hydrogen sulfide (H_2_S)-targeted treatment could serve as reprogramming strategies to avert cardiovascular disease [29], whether propionate enhancing the sulfur-reducing bacteria *Fusibacter* is critical in reducing the BP in concert with the H_2_S signaling pathway deserves further clarification. Another mechanism proposed behind the programming of hypertension is renal inflammation with a pathological characteristic of increased proinflammatory cytokines [21]. According to our data, propionate treatment reduced IL-1A, IL-2, and IL-6 in the CP group instead of the CKDP group. Hence, the inhibition of inflammatory cytokines is not a primary mechanism contributing to the protective effects of propionate against CKD-induced hypertension.

There are, however, several limitations that should be acknowledged. First, we mainly drew attention to the kidneys. Therefore, very little is known about whether other organs that control BP have a role in the beneficial effect of propionate against maternal CKD-induced hypertension. Second, we did not evaluate microbiota changes and SCFA levels in dams. A comparison between offspring and their mothers might offer more details on how the gut microbiota and the derived metabolites of the dams could impact their offspring. Third, only male offspring were enrolled in the current study. Whether sex differences exist in the programming effects of propionate warrants further evaluation. Furthermore, propionate administration has been reported to mitigate adenine-induced kidney damage [30]. Given that we did not measure renal function in mother rats, the protective action of propionate is due directly to the increase in its transfer to the offspring or through its preventing progression of CKD in mother rats, which would require further clarification. Last, currently, a simultaneous determination of all microbial metabolites in a single study has not been reported. Even with our study providing evidence for SCFAs in the maternal CKD model, the effect of propionate supplementation on other microbial metabolites is still largely unknown.

## 5. Conclusions

In conclusion, perinatal propionate supplementation reshapes the composition of gut microbiota and regulates propionate and its receptor to afford protection against maternal CKD-induced hypertension. A deeper understanding of the interconnection mechanisms between propionate and CKD underlying the developmental programming of hypertension deserves further in-depth research efforts. Moving forward, our findings are of value to allow perinatal gut microbiota-targeted therapy applied as potential reprogramming interventions for future clinical translation.

## Figures and Tables

**Table 1:** qPCR primer sequences.

Gene	5′ Primer	3′ Primer
GPR41	5 tcttcaccaccgtctatctcac 3	5 cacaagtcctgccaccctc 3
GPR43	5 ctgcctgggatcgtctgtg 3	5 cataccctcggccttctgg 3
GPR109A	5 cggtggtctactatttctcc 3	5 cccctggaatacttctgatt 3
Olfr78	5 gaggaagctcacttttggtttgg 3	5 cagcttcaatgtccttgtcacag 3
R18S	5 gccgcggtaattccagctcca 3	5 cccgcccgctcccaagatc 3

**Table 2:** Weights and blood pressure in offspring at 12 weeks of age.

Groups	C	CKD	CP	CKDP
Mortality	0%	0%	0%	0%
Body weight (BW) (g)	281 ± 8 ^a^	304 ± 18 ^a^	319 ± 11 ^a^	269 ± 7 ^b^
Left kidney weight (KW) (g)	1.31 ± 0.05 ^b^	1.62 ± 0.08 ^a^	1.40 ± 0.05 ^b^	1.33 ± 0.06 ^b^
Left KW/100 g BW	0.47 ± 0.01	0.55 ± 0.05	0.4 ± 0.01	0.49 ± 0.01
Systolic BP (mmHg)	132 ± 1 ^b^	144 ± 1 ^a^	133 ± 1 ^b^	135 ± 1 ^b^
Diastolic BP (mmHg)	87 ± 2	95 ± 2	86 ± 2	91 ± 1
Mean arterial pressure (mmHg)	102 ± 1 ^b^	112 ± 2 ^a^	101 ± 1 ^b^	106 ± 1 ^b^

N = 7–8/group; the letters ^a^ and ^b^ denote significant differences between groups (*p* < 0.05, one-way ANOVA). BP = blood pressure. C = control; CKD = adenine-treated rats; CP = control rats received propionate; CKDP = adenine-treated rats received propionate.

**Table 3:** Plasma SCFA levels in offspring at 12 weeks of age.

Groups	C	CKD	CP	CKDP
Acetic acid (μM)	401.4 ± 14.5	413.8 ± 18.1	425.6 ± 13.9	389.8 ± 17.7
Propionic acid (μM)	4.1 ± 0.27 ^b^	4.24 ± 0.37 ^b^	9.73 ± 0.34 ^a^	10.19 ± 0.55 ^a^
Isobutyric acid (μM)	4.48 ± 0.19	4.34 ± 0.13	4.55 ± 0.11	4.67 ± 0.15
Butyric acid (μM)	5.44 ± 0.66	5.54 ± 0.61	7.52 ± 1.03	4.98 ± 0.99
Isovaleric acid (μM)	6.98 ± 0.27	7.89 ± 0.29	7.08 ± 0.48	7.17 ± 0.42
Valeric acid (μM)	5.26 ± 0.42	5.79 ± 0.29	5.33 ± 0.63	5.75 ± 0.44

N = 7–8/group; the letters ^a^ and ^b^ show significant differences between groups (*p* < 0.05, one-way ANOVA). C = control; CKD = adenine-treated rats; CP = control rats received propionate; CKDP = adenine-treated rats received propionate.

## Data Availability

Data are contained within the article.
